# Validation of a telephone‐based administration of the simplified nutritional appetite questionnaire

**DOI:** 10.1002/jcsm.13264

**Published:** 2023-05-22

**Authors:** Binh Duong Thai, Jürgen M. Bauer, Annette Eidam, Jane Durga, Stefan Grund, Thomas Mross, Petra Benzinger

**Affiliations:** ^1^ Center for Geriatric Medicine Heidelberg University Hospital Heidelberg Germany; ^2^ Institute of Health and Generations Kempten University of Applied Sciences Kempten Germany

**Keywords:** Appetite, Assessment, Malnutrition, Older persons

## Abstract

**Background:**

Anorexia of aging is characterized by an age‐associated reduction of appetite, whose aetiology in most cases is multifactorial and which often triggers malnutrition. The Simplified Nutritional Appetite Questionnaire (SNAQ) is an established screening tool. This study aimed to investigate reliability, validity, and feasibility of its telephone administration (T‐SNAQ) in German community‐dwelling older adults.

**Methods:**

This cross‐sectional single‐centre study recruited participants from April 2021 to September 2021. First, the SNAQ was translated into German according to an established methodology. After translation, reliability, construct validity, and feasibility of the T‐SNAQ were analysed. A convenience sample of community‐dwelling older adults aged ≥70 years was recruited. The following measurements were applied to all participants: T‐SNAQ, Mini Nutritional Assessment – Short Form (MNA‐SF), six‐item Katz index of independence in activities of daily living (ADL), eight‐item Lawton instrumental activities of daily living (IADL), telephone Montreal Cognitive Assessment (T‐MoCA); FRAIL scale, Geriatric Depression Scale (GDS‐15) and Charlson co‐morbidity index as well as daily caloric and protein intake.

**Results:**

One hundred twenty participants (59.2% female) with a mean age of 78.0 ± 5.8 years were included in the present study. The percentage of participants identified with poor appetite based on T‐SNAQ was 20.8% (*n* = 25). T‐SNAQ showed a good internal reliability with a Cronbach's alpha coefficient of 0.64 and a good test–retest reliability [intraclass coefficient of 0.95 (*P* < 0.05)]. Regarding construct validity, T‐SNAQ was significantly positively correlated with MNA‐SF (*r* = 0.213), T‐MoCA (*r* = 0.225), daily energy (*r* = 0.222) and protein intake (*r* = 0.252) (*P* < 0.05). It also demonstrated a significant negative association with GDS‐15 (*r* = −0.361), FRAIL scale (*r* = −0.203) and Charlson co‐morbidity index (*r* = −0.272). Regarding applicability, the mean time for T‐SNAQ was 95 s and completion rate was 100%.

**Conclusions:**

The T‐SNAQ is a feasible screening instrument for anorexia of aging in community‐dwelling older adults via telephone interviews.

## Introduction

Aging is associated with degenerative processes in many organ systems such as the cardiovascular, respiratory, endocrine, musculoskeletal, and gastrointestinal system. For example, muscle mass decreases approximately by 0.7% per year in women and by 0.9% per year in men. As a consequence of reduced muscle mass and decreased physical activity, energy requirements in older adults decline [S1]. Therefore, older persons reduce their energy consumption. In a significant number of older persons, a maladaptive loss of appetite occurs that is often caused by co‐morbidities and pharmacotherapy and that may result in energy intake below energy requirement. This complex phenomenon has been described as anorexia of aging.^1^ The prevalence of anorexia of aging in community‐dwelling older people ranges from 15% to 30%.^2^

The mechanisms that influence an older person's appetite are complex involving physiological aging processes as well as coexistent disease and external factors.^3^ Relevant mechanisms include impaired chemosensory functions, gastrointestinal dysfunction, ineffective central regulation of appetite‐related hormones, psychosocial changes, and increase in medication use.^4^ Anorexia of aging is associated with a high risk of malnutrition.^5^ Malnutrition may lead to sarcopenia and frailty, both associated with negative health‐related events in older adults.^6^

A valid and reliable tool to early detect loss of appetite in older persons is therefore clearly needed. The ‘Appetite, Hunger and Sensory Perception Questionnaire (AHSP)’ is a comprehensive screening tool. Its 29 items address the feelings of hunger and appetite together with taste and smell perception.^7^ However, its length and complexity have hampered its widespread use.^8^ As a consequence, a simpler and easier to use appetite questionnaire, the Simplified Nutritional Appetite Questionnaire (SNAQ), was developed.^9^ The SNAQ was able to predict involuntary weight loss of at least 5% from baseline body weight within 6 months in community‐dwelling older adults.^9^ The SNAQ has been regarded as an efficient clinical screening tool, and it was assessed in different settings and patient populations.^10^ Over recent years, it has been translated to several languages.^11^, ^12^, ^13^, ^14^, ^15^, ^16^ Although the SNAQ has been already used in some studies in German‐speaking countries [S2‐S4], these versions have not been developed based on a methodology to allow for cross‐cultural adaptations. As a consequence, no validated German translation has been available.

COVID‐19 has been a particular threat to older persons causing significant morbidity and high mortality rates in this population [S5‐S7]. In addition, COVID‐19 in older patients has been associated with malnutrition.^17^ In a cross‐sectional study of COVID‐19 patients above age 65, 27.5% of the participants were at risk of malnutrition and 52.7% were categorized as malnourished.^18^ In addition, loss of appetite and smell were key symptoms for COVID‐19 infections, especially during the early phase of the pandemic.^19^ Screening for anorexia of aging may allow for early identification of COVID‐19 patients who are at an increased risk of malnutrition. Most likely, the application of video or telephone counselling in older patients will be significantly increased in the future. A telephone‐based screening tool may offer the advantage to allow for time‐efficient risk monitoring in this context.

This study aimed to assess the validity and reliability of a German version of the SNAQ administered through telephone interview (T‐SNAQ) in a convenience sample of community‐dwelling older people during the COVID‐19 pandemic.

## Methods

### Translation and cross‐cultural adaptation

The SNAQ is a self‐assessment questionnaire for evaluating appetite in older people. It comprises four items: appetite, satiety, taste of food, and number of meals eaten per day. Each item is rated on a scale between one and five. The total score ranges from 4–20, with higher scores indicating better appetite. A cut‐off ≤14 predicts involuntary weight loss.^9^ The translation process of the SNAQ from English to German was based on published recommendations.^20^ Briefly, the SNAQ was simultaneously translated into German by three independent researchers (two physicians experienced in geriatric medicine and one nutrition scientist). The translations were reviewed and discussed to reach a consensus draft version, which was translated back to English by a bilingual physician blinded to the original questionnaire. Subsequently, the back‐translation was sent to the original author to rule out semantic and conceptual inconsistencies between the original SNAQ and the back‐translated version. The draft‐version of the German SNAQ was tested in three older persons to verify its comprehensibility and the appropriateness. Minor modifications were suggested, and a final version was agreed upon (Figure S1).

### Participants

Community‐dwelling older people were recruited by word of mouth and advertisements in senior organizations and day centres. Participants were enrolled between April 2021 and September 2021. Eligibility criteria were (i) age ≥70 years, (ii) community‐dwelling, and (iii) availability of a scale to measure bodyweight. Exclusion criteria were any visual and auditory impairments that precluded assessments through telephone interview and paper‐and‐pencil questionnaires. The study was planned and conducted when contact restrictions were in place. Hence, there were no in‐person contacts throughout the study. Interested individuals received a telephone call and written information about the study. Eligible persons were asked to send back the signed consent form. We aimed to obtain at least 100 complete assessments [S8].

### Study design

Data in the study were collected through a telephone interview and paper‐and‐pencil questionnaires. Given the lack of in‐person contact, data collection was divided into four steps (Figure S2). (1) A questionnaire was sent out to participants asking for information on anthropomorphic and socio‐demographic data, Lawton instrumental activities of daily living (IADL), FRAIL scale (Fatigue, Resistance, Ambulation, Illnesses, & Loss of Weight), Geriatric Depression Scale with 15 items (GDS‐15), Charlson co‐morbidity index (age‐adjusted), and number of medications. Information was checked for completeness and plausibility upon arrival of the questionnaire at the study centre. Missing information was ascertained during a telephone call in step 2, if needed. (2) A telephone interview was carried out including T‐SNAQ_T0_, Montreal Cognitive Assessment (telephone‐version, T‐MoCA), Mini Nutritional Assessment –Short form (MNA‐SF), and Katz Index of Independence in Activities of Daily Living (Katz ADL index). (3) One to 3 days later, T‐SNAQ was again administered (T‐SNAQ_T1_). (4) Finally, the SNAQ (SNAQ_T2_) was sent out to participants along with a three‐day food diary and returned after completion.

### Data collection

To characterize the sample, information on age and gender was provided by participants. Functional status was assessed using Katz ADL index^21^ and Lawton and Brody's instrumental ADL (IADL) index.^21^, ^22^ To evaluate cognitive status, a telephone version of the Montreal Cognitive Assessment validated in a German population was carried out (T‐MoCA).^23^ Participants with a score of ≤17 were considered cognitively impaired.^24^ In order to describe the medical status, participants were asked to fill out the age‐adjusted Charlson co‐morbidity index^25^ and to list all current medications.

#### Nutritional assessment

Body mass index was calculated based on participants' self‐reported weight and height. MNA‐SF was used to evaluate the nutritional status. This questionnaire is highly sensitive and specific for malnutrition screening.^26^ Community‐dwelling older adults with poor appetite might demonstrate lower consumption of energy and protein.^27^ Daily dietary caloric and macronutrients intake were assessed with a self‐reported 3‐day food diary. To increase compliance, participants were asked to estimate portion sizes rather than to weigh portions. After receiving the completed food diaries, a quality control was performed, and intake was estimated using DGExpert (https://www.dgexpert.de, version 2.0, developed and provided by the German Nutrition Society and based on the German Nutrient Data Base Version 3.02).

#### Convergent validity

Next to nutritional parameters further assessments were chosen to test convergent validity: (1) As reported in a cross‐sectional study of depression in geriatric patients, SNAQ was expected to correlate with depressive symptoms.^28^ Depressive symptoms in this study were assessed using the German version of the Geriatric Depression Scale (GDS‐15) with a cut‐off value of ≥5 to detect depressive symptoms.^29^ (2) Although scales and methods were assessed differently, previous studies showed that decline in appetite was associated with an increased risk of frailty in older adults.^30^, ^31^ FRAIL scale is a simple screening tool for frailty in older adults. The questionnaire includes five items: fatigue, resistance, ambulation, illness, and loss of weight. Participants were classified into three categories according to their score on the FRAIL scale: robust (0 points), prefrail (1–2 points), and frail (≥3 points).^32^ (3) Anorexia of aging is more prevalent in older adults with a high co‐morbidity load.^2^ In order to capture the multimorbidity of participants, the age‐adjusted Charlson co‐morbidity index was used in this study.^25^

#### Divergent validity

T‐MoCA, education as assessed by years of formal education, and Katz ADL index were expected not to correlate with anorexia of aging.

### Statistical analysis

Descriptive data were presented as mean ± standard deviation or percentages depending on their structure and distribution.

1. Reliability:
  Test–retest reliability was determined by calculating the intraclass correlation coefficient (ICC) with 95% confidence interval (95% CI) of T‐SNAQ_T0_ and T‐SNAQ_T1_. To determine the reliability of SNAQ assessed during telephone interview, ICC of T‐SNAQ_T0_ and the paper‐and‐pencil version SNAQ_T2_ was calculated. ICC with values <0.5, between 0.5 and 0.75, between 0.75 and 0.9, and >0.90 are representative for poor, moderate, good, and excellent reliability [S9].
  To analyse the internal consistency of the T‐SNAQ_T0_, we used Cronbach's alpha coefficient. Factor analysis with Principal component analysis extraction method including varimax rotation was performed to identify the dimensionality of the German T‐SNAQ. Eigenvalues > 1 (Kaiser criteria) were accepted and items with a factorial loading of <0.4 were not showed.
2. Validity:
  Construct validity of T‐SNAQ_T0_ was assessed with convergent (MNA‐SF, energy and protein intake, FRAIL scale, Charlson co‐morbidity scale, and GDS‐15) and divergent validity (T‐MoCA, years of education, and Katz ADL index) analysis using Spearman's rho.
3. Feasibility:
  Feasibility of T‐SNAQ_T0_ was analysed using mean time of interview duration and completion rate.

All statistical tests were two‐tailed, with *P* ≤ 0.05 considered statistically significant. IBM SPPS Statistics version 27 for Windows (IBM Corp., Armonk, NY, USA) was used for statistical analysis.

## Results

### Study population

A total of 120 community‐dwelling older people participated in the study. Of these, 110 study participants provided valid food charts. The sample included 71 (59.2%) women and 49 (40.8%) men, with a mean age (SD) of 78.0 ± 5.84 and a mean BMI (SD) of 24.9 ± 3.55. Thirty‐five study participants were categorized as being malnourished or as being at risk of malnutrition according to the MNA‐SF. The characteristics of the study population are presented in Table 1. The German T‐SNAQ _T0_ had a mean score (SD) of 15.7 ± 2.14 with a minimum of 7 and a maximum of 19, when administered via telephone.

**Table 1:** Characteristics of the study sample (total sample *n* = 120)

Variables	Value
Age (years), mean (SD)	78.0 (5.8)
Female, *n* (%)	71 (59.2)
Katz index of ADL (score, 0–6), mean (SD)	5.8 (0.6)
IADL (score, 0–8), mean (SD)	
Male	7.4 (1.3)
Female	7.8 (0.8)
T‐MoCA (score, 0–22), mean (SD)	18.1 (2.5)
T‐MoCA ≤17, *n* (%)	41 (34.2)
Charlson co‐morbidity index (Age‐adjusted), mean (SD)	4.9 (2.1)
Number of medications, mean (SD)	4.7 (3.8)
BMI (kg/m^2^), mean (SD)	24.9 (3.55)
BMI ≥ 30, *n* (%)	12 (10.0)
25 ≤ BMI ≤ 29, *n* (%)	39 (32.5)
20 ≤ BMI ≤ 24, *n* (%)	62 (51.7)
BMI ≤ 19, *n* (%)	7 (5.8)
T‐SNAQ (score, 4–20), mean (SD)	15.7 (2.1)
T‐SNAQ ≤ 14, *n* (%)	25 (20.8)
MNA‐SF (score, 0–14), mean (SD)	12.1 (2.21)
Risk of malnutrition (8 ≤ score ≤ 11), *n* (%)	30 (25.0)
Malnutrition (score ≤7), *n* (%)	5 (4.2)
Daily caloric intake (kcal) (*n* = 110), mean (SD)	
Male	2193.8 (736.3)
Female	2006.3 (527.6)
Daily protein intake (g) (*n* = 110), mean (SD)	82.8 (27.9)
GDS‐15 (score, 0–15), mean (SD)	1.9 (2.6)
FRAIL scale (score, 0–5), mean (SD)	0.5 (0.97)
Pre‐frail, *n* (%)	30 (25.0)
Frail, *n* (%)	8 (6.7)
Education (years), mean (SD)	15.4 (3.2)

BMI, body mass index; FRAIL scale, Fatigue, Resistance, Ambulation, Illnesses, & Loss of Weight (higher scores indicating lower level of frailty); GDS‐15, Geriatric Depression Scale with 15 items (higher scores indicating higher level of depressive symptoms); IADL, Lawton instrumental activities of daily living (higher scores indicating higher level of independence); Katz index of ADL, Katz Index of Independence in Activities of Daily Living Scale (higher scores indicating higher level of independence); MNA‐SF, Mini Nutritional Assessment – Short Form (higher scores indicating lower risk for malnutrition); T‐MoCA, Montreal Cognitive Assessment (telephone version) (higher scores indicating better cognition); T‐SNAQ, Simplified Nutritional Appetite Questionnaire (telephone‐based) (higher scores indicating better appetite).

### Reliability

The ICC for test–retest reliability between T‐SNAQ_T0_ and T‐SNAQ_T1_ was 0.95 (95% CI 0.92 to 0.96). The interval between the two telephone interviews (T0 and T1) was 1.2 ± 0.54 days. T‐SNAQ_T0_ compared with SNAQ_T2_ demonstrated ICC of 0.92 (95% CI 0.88 to 0.94). The mean time interval (SD) between the first telephone interview (T0) and the self‐administration of the questionnaire (T2) was 7.4 ± 6.0 days. Subgroup analyses did not demonstrate any significant differences between participants with cognitive impairment and those without cognitive impairment according to the T‐MoCA (Table 2). For internal consistency, Cronbach's alpha of T‐SNAQ_T0_ was 0.64 (Table 2).

**Table 2:** Scores, Cronbach's alpha, and ICC of the T‐SNAQ for the total population, subgroups of participants with probable cognitive impairment (T‐MoCA ≤ 17, *n* = 41) and with intact cognition (T‐MoCA > 17, *n* = 79)

	Mean (SD)	Minimum	Maximum	Cronbach's alpha	ICC between T_0_ and T_1_ (95% CI)	ICC between T_0_ and T_2_ (95% CI)
Total population	15.68 (2.14)	7	19	0.64	0.95 (0.92–0.96)	0.92 (0.88–0.94)
T‐MoCA ≤ 17	15.17 (2.05)	10	19	0.58	0.91 (0.83–0.95)	0.93 (0.86–0.96)
T‐MoCA > 17	15.94 (2.16)	7	19	0.66	0.96 (0.94–0.98)	0.91 (0.86–0.94)

The factorial analysis with a Kaiser–Meyer–Olkin measure of sampling adequacy of 0.662 and a significant Bartlett's test of sphericity (*P* < 0.001, *df* = 6) showed that the German T‐SNAQ has two dimensions. Two factors (Factor 1: Appetite and Satiety; Factor 2: Meal Frequency) were identified with eigenvalues >1.00, which contributed to 76.4% of the total variance in the total score. Factorial loadings for two dimensions ranged between 0.78 and 0.99 (Table 3).

**Table 3:** Mean, standard deviation, minimum and maximum score, as well as factorial loading with total variance for each question of the T‐SNAQ

Question	Mean	SD	Minimum	Maximum	Factor 1: Appetite and satiety	Factor 2: Meal frequency
1	3.98	0.97	1	5	0.87	
2	3.61	0.70	1	5	0.78	
3	4.26	0.81	1	5	0.83	
4	3.83	0.54	3	5		0.99
Variance Cumulated %					51.1%	25.2%

Extraction method: Principal component analysis. Factorial loading values of <0.4 are not displayed in the table.

### Validity

The T‐SNAQ_T0_ demonstrated significant correlations with MNA‐SF, daily caloric and protein intake, T‐MoCA, GDS‐15, FRAIL scale and Charlson co‐morbidity index. It did not significantly correlate with years of education and Katz ADL index (Table 4).

**Table 4:** Spearman correlation (*r*_s_) between T‐SNAQ and other parameters

Variables	*r*_s_	*P*‐value
MNA‐SF	0.213	0.02
Caloric intake	0.222	0.02
Protein intake	0.252	0.008
GDS‐15	−0.361	<0.001
FRAIL scale	−0.203	0.026
Charlson Comorbidity Index	−0.272	0.003
T‐MoCA	0.225	0.014
Years of education	0.017	0.858
Katz index of ADL	0.100	0.277

FRAIL scale, Fatigue, Resistance, Ambulation, Illnesses, & Loss of Weight (higher scores indicating lower level of frailty); GDS‐15, Geriatric Depression Scale with 15 items (higher scores indicating higher level of depressive symptoms); Katz index of ADL, Katz Index of Independence in Activities of Daily Living Scale (higher scores indicating higher level of independence); MNA‐SF, Mini Nutritional Assessment – Short Form (higher scores indicating lower risk for malnutrition); T‐MoCA, Montreal Cognitive Assessment (telephone version) (higher scores indicating better cognition).

### Feasibility

The mean duration for telephone administration of the T‐SNAQ_T0_ was 1.6 min ± 0.7 and 1.3 min ± 0.5 in the first and second assessment, respectively. The completion rate of the two assessments was 100%.

## Discussion

This is the first study to analyse the reliability, validity, and feasibility of the German SNAQ as a telephone‐based screening (T‐SNAQ) in a convenience sample of community‐dwelling older adults. The prevalence of anorexia of aging in this sample was comparable with prevalence rates for this population in the literature, ranging between 15% and 30%.^2^ Test–retest reliability for the telephone assessment was excellent as was reliability between telephone assessment and self‐administration. Subgroup analyses demonstrated that the SNAQ was well suited for older adults with mild cognitive impairment as well.

Cronbach's alpha coefficient in this study can be considered as acceptable for preliminary and basic research [S10]. However, it has to be considered that Cronbach's alpha coefficient is influenced by the number of items. A questionnaire with fewer items is therefore expected to be associated with a lower value [S11]. Previous studies that validated the SNAQ demonstrated similar results with Cronbach's alpha coefficients ranging between 0.50 and 0.75.^11^, ^12^, ^13^, ^16^

Unlike some other validation studies, we found T‐SNAQ to have two factors, which were first ‘appetite and satiety’ and second ‘meal frequency’. Two previous studies also showed that the SNAQ had more than one factor.^33^, ^34^ In the present study, food perception, food intake and satiety explained most of the variance. The second factor ‘food frequency’ might reflect eating habits of older community‐dwelling adults in Germany.

With regard to validity, the T‐SNAQ was significantly associated (*P* ≤ 0.05) with the MNA‐SF, daily caloric and protein intake, the GDS‐15, the FRAIL scale, and the Charlson co‐morbidity index. Similar correlations were reported in validation studies for the SNAQ in Japanese and Turkish.^11^, ^12^ Correlation between the MNA long form and SNAQ was reported to be higher than the correlation we found between MNA‐SF and T‐SNAQ in our study.^34^ However, it has to be considered that SNAQ and MNA‐SF measure similar traits but that they are based on different constructs. While the main focus of SNAQ is poor appetite, MNA‐SF was developed with the aim to identify older persons with malnutrition and those who are at risk of malnutrition by using parameters such as eating difficulties, weight loss in the past, mobility, stress or illness, dementia or depression, and BMI.^9^, ^35^ Hence, criteria of the MNA‐SF also reflect longer‐standing conditions such as cognition and depressive symptoms. In contrast, appetite is influenced not only by the fasting between meals but also by external factors like the sensory attractiveness of nicely prepared dishes.^36^ As a consequence, appetite can be expected to show relevant fluctuation from day to day and even within single days which may explain, at least in part, the low correlation between both constructs.

We also observed a significant positive correlation between the T‐SNAQ and the daily caloric as well as the protein intake thereby confirming previous findings.^13^ Our data show that there is a significant association between poor appetite and a decrease in total energy and protein intake the latter illustrating the increased risk of malnutrition in older adults with poor appetite.^5^ Although our findings are cross‐sectional, they strongly support the assumption that screening of appetite by SNAQ identifies persons at risk for malnutrition. The construct validity of the T‐SNAQ was further supported by its correlation with depressive symptoms, frailty and co‐morbidity. The correlation between T‐SNAQ and GDS‐15 was in the same range as reported by others for the SNAQ.^11^ In the present study, we confirmed that loss of appetite in community‐dwelling older adults correlated moderately with presence of frailty, the later having been screened for by the FRAIL scale. The correlations with the Charlson co‐morbidity index was in line with findings of medical conditions such as gastrointestinal disease, malabsorption syndromes, acute and chronic infection, hypermetabolism, as well as cancer and rheumatoid arthritis all causing anorexia.^37^

In contrast to our expectations, T‐SNAQ correlated significantly with the T‐MoCA score. Although a high prevalence of malnutrition in older adults with dementia has been reported,^38^ information has been scarce on the association between appetite and cognitive impairment. It has been demonstrated that olfactory dysfunction may be present in patients with mild cognitive impairment and also in different types of dementia.^39^ Taking these observations into account, the decrease of appetite in persons with cognitive impairment may be regarded as plausible.^3^ Our findings should trigger additional research on appetite loss in older persons with early cognitive decline.

With regard to feasibility, the T‐SNAQ could be easily and quickly administered. As a consequence of having become familiar with the questionnaire its administration was further accelerated when the first and the second screening were compared.

When interpreting the results of the present study, several limitations have to be considered. First, our validation study recruited community‐dwelling older adults only. Validation studies should be done in other populations of older adults in the future. Second, physical performance was not assessed in this study due to the pandemic situation. Moreover, all assessments were either self‐rated or based on telephone interviews. In particular, BMI was calculated based on self‐report and MNA‐SF was self‐rated. Agreement between MNA‐SF administered by health care professionals and self‐rated MNA has been reported to be excellent by others [S12]. Yet, comparing results between different studies, self‐reported BMI as opposed to direct measurement of height and weight might have influenced the correlation between MNA‐SF and SNAQ. Furthermore, due to the study design, we were not able to test for COVID‐19 at the time of interviews in order to exclude COVID‐19 infection as a potential bias. However, infection rates were rather low at that specific time period and none of the participants mentioned any febrile condition during the interviews or reported on medication typically used to treat febrile infections. Finally, we did not evaluate the ability of the T‐SNAQ to predict future weight loss in our study participants.

In conclusion, the German T‐SNAQ proved to be a simple and reliable instrument for distant assessment of appetite in community‐dwelling older adults. Further studies of the German T‐SNAQ should be conducted to confirm its predictive validity.

## Conflict of interest statement

Binh Duong Thai declares that he has no conflict of interest. Jürgen M. Bauer received consultation fees from Nestlé, Danone Nutricia, and Rejuvenate Biomed. Jürgen M. Bauer received payment for lectures from Novartis, Pfizer, Nestlé, Danone Nutricia, Fresenius, UCB Pharma, Daiichi Sankyo, and Bayer. Jürgen M. Bauer is a board member of the European Geriatic Medicine Society. Annette Eidam declares that she has no conflict of interest. Jane Durga received consultation fess from Fresenius Kabi. Stefan Grund declares that he has no conflict of interest. Thomas Mross declares that he has no conflict of interest. Petra Benzinger declares that she has no conflict of interest.

## Supporting information


**Data S1.** Supporting Information

**Figure S1.** The Simplified Nutritional Appetite Questionnaire in German.
**Figure S2.** Study design.
