# Supplementary material for: Validation of a telephone‐based administration of the simplified nutritional appetite questionnaire
Source: J Cachexia Sarcopenia Muscle. 2023 May 22;14(4):1848–54. doi: 10.1002/jcsm.13264 (PMC10401531; doi:10.1002/jcsm.13264)
Supplement: Supplementary file 1 — Data S1. Supporting Information [file JCSM-14-1848-s002.docx]

**Online Supplement for additional References**

1. Speakman JR, Westerterp KR (2010) Associations between energy demands, physical activity, and body composition in adult humans between 18 and 96 y of age. Am J Clin Nutr 92:826–834. <https://doi.org/10.3945/ajcn.2009.28540>
2. Pourhassan M, Sieske L, Janssen G et al. (2020) The impact of acute changes of inflammation on appetite and food intake among older hospitalised patients. Br J Nutr 124:1069–1075. https://doi.org/10.1017/S0007114520002160
3. Helfenstein SF, Uster A, Rühlin M et al. (2016) Are Four Simple Questions Able to Predict Weight Loss in Outpatients With Metastatic Cancer? A Prospective Cohort Study Assessing the Simplified Nutritional Appetite Questionnaire. Nutr Cancer 68:743–749. https://doi.org/10.1080/01635581.2016.1180412
4. Sieske L, Janssen G, Babel N et al. (2019) Inflammation, Appetite and Food Intake in Older Hospitalized Patients. Nutrients 11. https://doi.org/10.3390/nu11091986
5. Kunz R, Minder M (2020) COVID-19 pandemic: palliative care for elderly and frail patients at home and in residential and nursing homes. Swiss Med Wkly 150:w20235. https://doi.org/10.4414/smw.2020.20235
6. Wang W, Tang J, Wei F (2020) Updated understanding of the outbreak of 2019 novel coronavirus (2019-nCoV) in Wuhan, China. J Med Virol 92:441–447. https://doi.org/10.1002/jmv.25689
7. Silverio R, Gonçalves DC, Andrade MF et al. (2021) Coronavirus Disease 2019 (COVID-19) and Nutritional Status: The Missing Link? Adv Nutr 12:682–692. <https://doi.org/10.1093/advances/nmaa125>
8. Anthoine E, Moret L, Regnault A et al. (2014) Sample size used to validate a scale: a review of publications on newly-developed patient reported outcomes measures. Health Qual Life Outcomes 12:176. https://doi.org/10.1186/s12955-014-0176-2
9. Koo TK, Li MY (2016) A Guideline of Selecting and Reporting Intraclass Correlation Coefficients for Reliability Research. J Chiropr Med 15:155–163. <https://doi.org/10.1016/j.jcm.2016.02.012>
10. Peterson RA (1994) A Meta-Analysis of Cronbach's Coefficient Alpha. J CONSUM RES 21:381. <https://doi.org/10.1086/209405>
11. Bland JM, Altman DG (1997) Cronbach's alpha. BMJ 314:572. <https://doi.org/10.1136/bmj.314.7080.572>
12. Huhmann MB, Perez V, Alexander DD et al. (2013) A self-completed nutrition screening tool for community-dwelling older adults with high reliability: a comparison study. J Nutr Health Aging 17:339–344. https://doi.org/10.1007/s12603-013-0015-x
