# Supplementary material for: Validation of a telephone‐based administration of the simplified nutritional appetite questionnaire
Source: J Cachexia Sarcopenia Muscle. 2023 May 22;14(4):1848–54. doi: 10.1002/jcsm.13264 (PMC10401531; doi:10.1002/jcsm.13264)
Supplement: Supplementary file 2 — Figure S1. The Simplified Nutritional Appetite Questionnaire in German. Figure S2. Study design. [file JCSM-14-1848-s001.docx]

**Online Supplementary Material**

Validation of a telephone-based administration of the Simplified Nutritional Appetite Questionnaire

Thai D^1^, Bauer, J.M.^1^, Eidam A^1^, Durga J^1^, Grund S^1^, Mross T^1^, Benzinger, P.^1,2^

^1^ Center for Geriatric Medicine, Heidelberg University,

Agaplesion Bethanien Krankenhaus

Rohrbacher Strasse 149

69126 Heidelberg

Germany

^2^ Institute of Health and Generations

University of Applied Sciences Kempten

Bahnhofstrasse 61

87435 Kempten

Germany

Corresponding author:

Petra Benzinger, MD

Phone: +49 62213191783

Email: [petra.benzinger@agaplesion.de](mailto:petra.benzinger@agaplesion.de)

| **THE SIMPLIFIED NUTRITIONAL APPETITE QUESTIONNAIRE** |
| --- |
| Name ______________, Geschlecht (umkreisen): männlich weiblich, Alter______, Gewicht______, Größe______, Datum________ |
| Hinweise zur Durchführung: Bitten Sie die Testperson, den Fragebogen durch Umkreisen der zutreffenden Antworten auszufüllen, und zählen Sie danach die Ergebnisse basierend auf der folgenden numerischen Skala zusammen: a=1, b=2, c=3, d=4, e=5. Die Summe der Punktzahlen für die einzelnen Items bildet den SNAQ-Score. Ein SNAQ-Score ≤14 zeigt ein signifikantes Risiko für einen Gewichtsverlust von mindestens 5 % innerhalb von sechs Monaten an. |
| ***1. Mein Appetit ist*** |
| a. sehr schlecht |
| b. schlecht |
| c. durchschnittlich |
| d. gut |
| e. sehr gut |
| Punktzahl: /5  Kommentar: |
| ***2. Wenn ich esse,*** |
| a. fühle ich mich satt, nachdem ich nur wenige Bissen gegessen habe. |
| b. fühle ich mich satt, nachdem ich etwa ein Drittel einer Mahlzeit gegessen habe. |
| c. fühle ich mich satt, nachdem ich mehr als die Hälfte einer Mahlzeit gegessen habe. |
| d. fühle ich mich satt, nachdem ich den größten Teil einer Mahlzeit gegessen habe. |
| e. fühle ich mich fast nie satt. |
| Punktzahl: /5  Kommentar: |
| ***3. Das Essen schmeckt*** |
| a. sehr schlecht |
| b. schlecht |
| c. durchschnittlich |
| d. gut |
| e. sehr gut |
| Punktzahl: /5  Kommentar: |
| ***4. Normalerweise esse ich*** |
| a. weniger als eine Mahlzeit pro Tag. |
| b. eine Mahlzeit pro Tag. |
| c. zwei Mahlzeiten pro Tag. |
| d. drei Mahlzeiten pro Tag. |
| e. mehr als drei Mahlzeiten pro Tag. |
| Punktzahl: /5  Kommentar:  Gesamtpunktzahl: /20 |

**Fig. 1 The Simplified Nutritional Appetite Questionnaire in German**

**Fig. 2 Study design**
